# Obstacles to an Effective Transition to Adult Services for Patients with Hirschsprung Disease

**DOI:** 10.3390/children11101237

**Published:** 2024-10-14

**Authors:** Joseph R. Davidson, Joe Curry

**Affiliations:** 1 UCL Great Ormond Street Institute of Child Health, London WC1N 1EH, UK; 2 Evelina London Children’s Hospital, London SE1 7EH, UK; 3 Great Ormond Street Hospital for Children, London WC1N 3JH, UK; joe.curry@gosh.nhs.uk

**Keywords:** Hirschsprung disease, HSCR, long-term outcomes, transitional care

## Abstract

A growing number of patients with Hirschsprung disease are reaching adulthood, of whom a significant minority will require ongoing input from healthcare providers. In order to ensure patients receive the best care possible, it is essential to transition patients appropriately to adult services. This article describes the unmet need and some of the obstacles to this process and explores potential solutions, drawing on model examples for transitional care.

## 1. Introduction

Survival to adulthood is expected in the vast majority of patients with Hirschsprung disease (HSCR) who do not have a syndromic association [1,2]. This marks a clear triumph in the management of a congenital colorectal disorder that would have resulted in certain death almost 150 years ago during the time of Harald Hirschsprung, the Danish physician from whom the condition takes its name (the initial description has been translated into English [3]). However, both the condition itself and the procedures used to treat it carry a burden of morbidity and abnormal bodily function that can last well into adult life, if not the patient’s entire life. This article aims to explore the aspects of life that ought to be considered in the clinical and holistic management of HSCR patients as they depart paediatric services and enter the realm of adult health care. It draws upon a number of recently published studies of transitional care, both in HSCR and for other congenital conditions more widely.

## 2. The Unmet Need: Functional and Quality of Life Impairments in Adulthood

The modern lens of paediatric surgical outcomes has shifted focus in the last decade towards the longer-term well-being of patients, with a consistent recognition that a significant minority of HSCR patients will suffer from sustained functional impairment [4,5,6]. Our own group has focused on the inter-relationship of functional domains and quality of life metrics, identifying that patients who do poorly will have some degree of both bladder and bowel dysfunction and that these patients are most likely to have an associated psychosocial impact as a result of their condition [4,7,8,9,10]. This emphasises the need for a protocolised and focused enquiry of symptoms across multiple systems and also for specific psychological support.

Although “good” bowel functional outcomes might be expected for the majority of patients, with most managing their limited symptoms with diet alone [11], around one in ten patients could be expected to require colonic irrigation or opt for diversion with an end-ostomy [12]. Additionally, our own evidence suggests a small proportion of patients deemed fine at the point of discharge from the paediatric surgical service may develop new onset symptoms in adulthood [13]. This is further supported by evidence from 

Finland, suggesting that patients in their fourth and fifth decade may have worse bowel function than younger adults [14]. 

Alongside the predicted bowel functional impairments that might be expected with a congenital colorectal disorder, a further recognised factor ought to be considered, which is that of sexual function and fertility. Fortunately, erectile function and other sexual functional outcomes in male patients appear to be broadly preserved, with poorer function seeming to, once more, be associated with the worst bowel functional outcome [15,16]. Fertility outcomes in male patients also appear to closely mirror those expected in the general population in terms of access and success utilising assisted fertility services [17]. In contrast, however, the sexual functional outcomes in female patients are notably inferior to those in the general population, as demonstrated in our recent multi-centre study [18]. Furthermore, there is a marked incidence of tubal adhesions and associated subfertility in female patients, who have been shown elsewhere to have fewer children, be older at their first child, and have a higher access to IVF services than the general population [19]. 

The aforementioned issues with female fertility and sexual function came to light through a survey-based study of female patients across a large patient population spanning several tertiary centres. We anticipate that there may be a considerable burden of other conditions in patients who have undergone surgery for Hirschsprung that may not come to the attention of medical professionals, such as haemorrhoids or rectal prolapse, or may come to light far later in life, such as colorectal cancer. Such morbidity might only be recognised if active enquiry occurs prospectively across a large population of patients, such as the recently established European Reference Networks (ERNs) [20].

A specific consideration also needs to be made for patients with associated learning disability, which is a substantial proportion of patients with HSCR, owing to the recognised association with several syndromes (most importantly Trisomy 21). We have identified that patients with these two concomitant conditions fair very poorly in the long run in terms of functional outcome, with a ten-fold higher use of end colostomy in adulthood [1,21,22]. This may not be surprising, given the early onset cognitive decline that is known to occur in Trisomy 21 [23,24,25]. What is interesting is the minimal impact on the quality of life that a stoma seems to have in such cases, compared to the considerable impact on measured quality of life indicators in patients with normal cognition [8]. We, therefore, believe that a permanent stoma should be considered a viable treatment option in any learning-disabled patient, especially in those who are not doing well after their pull-through.

## 3. Readiness of the Adult Healthcare System to Manage Patients with Hirschsprung

Several issues coincide with the time a patient with HSCR departs their paediatric care. Preparing for these and mitigating them where possible is clearly a crucial aspect of managing the transition to adult care services appropriately.

### 3.1. Lack of Specific Knowledge and Expertise in Adult Services

First and foremost, it is important to recognise that while paediatric centres may treat several new patients with Hirschsprung disease every year, many adult gastrointestinal physicians and surgeons may never have encountered a single patient with the condition. 

The majority of adult gastrointestinal physicians and surgeons will be working within a professional landscape which is driven by targets for cancer, and, therefore, it might be idealistic to presume that adult practitioners would be able to take on the requisite workload required to fully understand and treat a complex disorder such as Hirschsprung, where patients may have ongoing issues with functional disturbances, require bowel management regimes to be adapted, and even require surgical intervention.

A prevalent theme in our own qualitative work with adult Hirschsprung patients was that they often found adult bowel management specialists to have little awareness of how their condition may differ from others that they treat and, therefore, found treatment to be of limited effectiveness and were often frustrated by their interactions. 

### 3.2. Suggestions for Suitable Onward Referrals

Preparing an adult service to receive a young adult patient by providing appropriate and adequate information is undoubtedly important [26]. We would propose a model whereby care for adults with congenital colorectal disorders might also be centralised within regions, much as it has been for congenital heart disease in many countries. Where a condition like Hirschsprung may struggle in comparison to transitional care for another chronic disease like congenital heart disease or diabetes is in the complex multi-system nature of the impairment for which patients will require follow-up. This raises the question of which responsible clinician a patient should be referred to. 

It is important to recognise that there may be long-lasting physical and psychological impacts of the condition and treatment [27] and that without formally enquiring after these, many may go undetected. Our own experience has identified patients may end up requiring the expertise of gastroenterologists, colorectal and general surgeons, pelvic floor specialists and gynaecologists, and, therefore, it remains something of a challenge to identify the correct destination for a patient in the absence of a true hospital generalist in adult practice, as the paediatric general surgeon might be regarded in the children’s setting. We also feel that the splintering of multidisciplinary care in a paediatric setting to many different adult services must be avoided, as it is all too likely to result in a patient left despondent in the face of a fragmented system of care.

Certainly, the proposed model of a centralised multi-disciplinary service for complex conditions such as Hirschsprung has significant implications for the overall health system. Globally, there will undoubtedly be systems that adopt similar models for rare or complex conditions (such as in Scandinavia, where many centralised services are offered in major cities for patients across the country or even internationally [28]). Furthermore, a hospital setting where pediatric and adult services are already co-located would significantly aid the efficiency of this process, whereby a collegiate atmosphere between services could see adolescents and young adults through the difficult period of transition.

### 3.3. Lack of Patient Awareness and Self-Advocacy

A final and potentially even greater issue is that the time of transition from paediatric care also represents a transition to independence in other aspects of life; throughout most childhood, the majority of patients will defer any and all decision-making and information about their condition to a parent or caregiver. This can, in many cases, lead to a young adult who is uninformed about their medical and surgical history as well as the nature of their condition [29]. Clearly, it is of paramount importance for a transition process to involve emphasising the role of patient education so that they are able to navigate their new horizons in the same way their parents had done in their childhood and adequately advocate for themselves and their health care. This process also requires concordance from parents, many of whom may be unaware of the need to relinquish control to their adolescent child in order that they might take on this new role [30].

## 4. Conclusions: How Best to Approach the Transition Process?

A statement often repeated in the field of transitional care is that it represents a process and not a point in time. Many effective models have been proposed [31]. However, all of these celebrate a gradual process that is tailored to the individual needs of a specific patient and associated family unit. This will usually involve discussion of the process from late childhood, encouragement of patient-centred learning and inclusion in medical decision-making, using joint appointments where the new care team might be introduced in a more familiar environment, and providing a safety net for the initial period of transition.

We believe that the advent of electronic medical records and patient access to these can deliver what we would call a “patient passport”, wherein relevant aspects of their condition and medical history can be summarised for the benefit of medical professionals. There have also been efforts to develop handbooks that outline specific considerations for patients with other rare diseases produced by patient organisations and provide key, relevant information for their ongoing management [32]. We have also introduced a formal assessment utilising standardised tools in order to identify those patients who may be particularly in need of routine ongoing appointments, as well as those who may require a single meeting to familiarise themselves with the process of navigating care in the adult healthcare setting.

## References

[1] Davidson J.R., Kyrklund K., Eaton S., Pakarinen M.P., Thompson D., Blackburn S.C., Cross K., De Coppi P., Curry J. (2021). Outcomes in Hirschsprung’s with coexisting learning disability. Eur. J. Pediatr..

[2] Neuvonen M.I., Kyrklund K., Lindahl H.G., Koivusalo A.I., Rintala R.J., Pakarinen M.P. (2015). A population-based, complete follow-up of 146 consecutive patients after transanal mucosectomy for Hirschsprung disease. J. Pediatr. Surg..

[3] Hirschsprung H. (1981). Constipation in the newborn as a result of dilation and hypertrophy of the colon. Dis. Colon. Rectum.

[4] Granström A.L., Danielson J., Husberg B., Nordenskjöld A., Wester T. (2015). Adult outcomes after surgery for Hirschsprung’s disease: Evaluation of bowel function and quality of life. J. Pediatr. Surg..

[5] Drissi F., Meurette G., Baayen C., Wyart V., Cretolle C., Guinot A., Podevin G., Lehur P.-A., Drissi F., Meurette G. (2019). Long-term Outcome of Hirschsprung Disease: Impact on Quality of Life and Social Condition at Adult Age. Dis. Colon. Rectum.

[6] Conway S.J., Craigie R.J., Cooper L.H., Turner K., Turnock R.R., Lamont G.L., Newton S., Baillie C.T., Kenny S.E. (2007). Early adult outcome of the Duhamel procedure for left-sided Hirschsprung disease—A prospective serial assessment study. J. Pediatr. Surg..

[7] Davidson J.R., Kyrklund K., Eaton S., Pakarinen M.P., Thompson D.S., Cross K., Blackburn S.C., De Coppi P., Curry J. (2021). Long-term surgical and patient-reported outcomes of Hirschsprung’s Disease. J. Pediatr. Surg..

[8] Hartman E.E., Oort F.J., Aronson D.C., Hanneman M.J., van Heurn E., de Langen Z.J., Madern G.C., Rieu P.N., van der Zee D.C., Looyaard N. (2007). Explaining Change in Quality of Life of Children and Adolescents With Anorectal Malformations or Hirschsprung Disease. Pediatrics.

[9] Byström C., Östlund S., Hoff N., Wester T., Granström A.L. (2020). Evaluation of Bowel Function, Urinary Tract Function, and Quality of Life after Transanal Endorectal Pull-Through Surgery for Hirschsprung’s Disease. Eur. J. Pediatr. Surg..

[10] Hartman E.E., Oort F.J., Aronson D.C., Hanneman M.J.G., van der Zee D.C., Rieu P.N.M.A., Madern G.C., De Langen Z.J., van Heurn L.W.E., van Silfhout-Bezemer M. (2004). Critical factors affecting quality of life of adult patients with anorectal malformations or Hirschsprung’s disease. Am. J. Gastroenterol..

[11] Lindert J. (2024). Influence of diet on bowel function and abdominal symptoms in children and adolescents with Hirschsprung Disease—A multinational patient-reported outcome survey. Children.

[12] Davidson J.R., Mutanen A., Salli M., Kyrklund K., De Coppi P., Curry J., Eaton S., Pakarinen M.P. (2022). Comparative cohort study of Duhamel and endorectal pull-through for Hirschsprung’s disease. BJS Open.

[13] Thompson D.S.M., Davidson J.R.M., Ford K.M., Loukogeorgakis S., Eaton S., Blackburn S.M., Curry J.M. (2024). Transitional Care In Patients With Hirschsprung Disease: Those Left Behind. Dis. Colon. Rectum.

[14] Jarvi K.M., Laitakari E.M., Koivusalo A., Rintala R.J., Pakarinen M.P. (2010). Bowel function and gastrointestinal quality of life among adults operated for Hirschsprung disease during childhood: A population-based study. Ann. Surg..

[15] Trinidad S., Garrison A., Encisco E.M., Canteria R., VanderBrink B., Strine A., Reddy P., Kotagal M., Rosen N., Rymeski B. (2023). Long-Term Male Sexual Function and Fecal Incontinence Outcomes for Adult Patients with Hirschsprung Disease or Anorectal Malformation. J. Pediatr. Surg..

[16] Davidson J.R., Eaton S., Pakarinen M.P., Curry J. (2023). Letter to the Editor in Response to: Long-term Male Sexual Function and Fecal Incontinence Outcomes for Adult Patients with Hirschsprung Disease or Anorectal Malformation. J. Pediatr. Surg..

[17] Davidson J.R., Kyrklund K., Eaton S., Pakarinen M.P., Thompson D.S., Cross K., Blackburn S.C., De Coppi P., Curry J. (2021). Sexual function, quality of life, and fertility in women who had surgery for neonatal Hirschsprung’s disease. Br. J. Surg..

[18] Davidson J.R., Mutanen A., Granström A.L., Hoel A.T., Loukogeorgakis P.S., De Coppi P., Bjørnland K., Wester T., Eaton S., Pakarinen P.M. (2024). Impaired Fertility and Sexual Function in Women with Hirschsprung disease: Results from an international multi-centre cross-sectional study. Authorea.

[19] Byström C., Örtqvist L., Gunnarsdóttir A., Wester T., Granström A.L. (2023). Fertility in patients with Hirschsprung’s disease: Population-based cohort study. BJS Open.

[20] Anzelewicz S.M., Petersen C., Czauderna P., Wijnen R. (2017). European Reference Networks: Share, Care, and Cure—Future or Dream?. Eur. J. Pediatr. Surg..

[21] Hackam D.J., Reblock K., Barksdale E.M., Redlinger R., Lynch J., A Gaines B. (2003). The influence of Down’s syndrome on the management and outcome of children with Hirschsprung’s disease. J. Pediatr. Surg..

[22] Travassos D., van Herwaarden-Lindeboom M., van der Zee D.C. (2011). Hirschsprung’s disease in children with Down syndrome: A comparative study. Eur. J. Pediatr. Surg..

[23] Niemczyk J., von Gontard A., Equit M., Medoff D., Wagner C., Curfs L. (2017). Incontinence in persons with Down Syndrome. Neurourol. Urodyn..

[24] Menezes M., Puri P. (2005). Long-term clinical outcome in patients with Hirschsprung’s disease and associated Down’s syndrome. J. Pediatr. Surg..

[25] Grieco J., Pulsifer M., Seligsohn K., Skotko B., Schwartz A. (2015). Down syndrome: Cognitive and behavioral functioning across the lifespan. Am. J. Med. Genet. C Semin. Med. Genet..

[26] Cairo S.B., Chiu P.P., Dasgupta R., Diefenbach K.A., Goldstein A.M., Hamilton N.A., Lo A., Rollins M.D., Rothstein D.H. (2018). Transitions in care from pediatric to adult general surgery: Evaluating an unmet need for patients with anorectal malformation and Hirschsprung disease. J. Pediatr. Surg..

[27] Hoel A.T., Tofft L., Bjørnland K., Gjone H., Teig C.J., Øresland T., Stenström P., Andersen M.H. (2021). Reaching adulthood with Hirschsprung’s disease: Patient experiences and recommendations for transitional care. J. Pediatr. Surg..

[28] Bjørland K., Qvist N., Wester T., Pakarinen M. (2017). Centralized Pediatric Surgery in the Nordic Countries: A Role Model for Europe?. Eur. J. Pediatr. Surg..

[29] Vargas M.C., Wehrli L.A., Louiselle A., Ketzer J., Reppucci M.L., Juddy-Glossy L., Alaniz V.I., Wilcox D.T., Wood D.N., Peña A. (2022). Do adult patients with congenital colorectal conditions know their diagnosis?. Pediatr. Surg. Int..

[30] Nah S.A., Ong C.C.P., Lie D., Marimuttu V.J., Hong J., Te-Lu Y., Low Y., Jacobsen A.S., Nah S.A., Ong C.C.P. (2018). Understanding Experiences of Youth Growing Up with Anorectal Malformation or Hirschsprung’s Disease to Inform Transition Care: A Qualitative In-Depth Interview Study. Eur. J. Pediatr. Surg..

[31] Royal College of Paediatrics and Child Health Best Practice Examples of Health Transition. Best Practice Examples of Health Transition 2024. https://www.rcpch.ac.uk/resources/best-practice-examples-health-transition.

[32] Love C., TOFS Charity Adult OA/TOF Management Handbook. Adult OA/TOF Management Handbook 2022. https://tofs.org.uk/oa-tof-information/professionals/adult-oa-tof-management-handbook/.

